# Woodland strawberry axillary bud fate is dictated by a crosstalk of environmental and endogenous factors

**DOI:** 10.1093/plphys/kiab421

**Published:** 2021-09-01

**Authors:** Javier Andrés, Julie Caruana, Jiahui Liang, Samia Samad, Amparo Monfort, Zhongchi Liu, Timo Hytönen, Elli A Koskela

**Affiliations:** 1 Department of Agricultural Sciences, Faculty of Agriculture and Forestry, University of Helsinki, Finland; 2 Department of Cell Biology and Molecular Genetics, University of Maryland, Maryland 20742, USA; 3 American Society for Engineering Education, Washington, District of Columbia, USA; 4 Department of Fruit Science, College of Horticulture, China Agricultural University, China; 5 Department of Biosystems and Technology, Swedish University of Agricultural Sciences, Alnarp SE-230 53, Sweden; 6 Centre for Research in Agricultural Genomics (CRAG), CSIC-IRTA-UAB-UB, 08193 Bellaterra, Barcelona, Spain; 7 Institut de Recerca i Tecnologia Agroalimentàries (IRTA), 08193 Barcelona, Spain; 8 NIAB East Malling Research, West Malling, ME19 6BJ, UK

## Abstract

Plant architecture is defined by fates and positions of meristematic tissues and has direct consequences on yield potential and environmental adaptation of the plant. In strawberries (*Fragaria vesca* L. and *F*. × *ananassa* Duch.), shoot apical meristems can remain vegetative or differentiate into a terminal inflorescence meristem. Strawberry axillary buds (AXBs) are located in leaf axils and can either remain dormant or follow one of the two possible developmental fates. AXBs can either develop into stolons needed for clonal reproduction or into branch crowns (BCs) that can bear their own terminal inflorescences under favorable conditions. Although AXB fate has direct consequences on yield potential and vegetative propagation of strawberries, the regulation of AXB fate has so far remained obscure. We subjected a number of woodland strawberry (*F. vesca* L.) natural accessions and transgenic genotypes to different environmental conditions and growth regulator treatments to demonstrate that strawberry AXB fate is regulated either by environmental or endogenous factors, depending on the AXB position on the plant. We confirm that the *F. vesca GIBBERELLIN20-oxidase4* (*FvGA20ox4*) gene is indispensable for stolon development and under tight environmental regulation. Moreover, our data show that apical dominance inhibits the outgrowth of the youngest AXB as BCs, although the effect of apical dominance can be overrun by the activity of *FvGA20ox4*. Finally, we demonstrate that the *FvGA20ox4* is photoperiodically regulated via *FvSOC1* (*F. vesca SUPPRESSOR OF OVEREXPRESSION OF CONSTANS1*) at 18°C, but at higher temperature of 22°C an unidentified *FvSOC1*-independent pathway promotes stolon development.

## Teaser

Environmental conditions and apical dominance dictate woodland strawberry plant architecture by regulating axillary bud fate.

## Introduction

Plant architecture is defined by meristem fates that can take on different avenues, depending on plant species and the position of the meristem. Shoot apical meristems (SAMs) can either remain vegetative and keep on producing new vegetative plant tissues, or, when induced to flower, differentiate into an inflorescence meristem. Axillary meristems (AXMs) are initiated in leaf axils and form axillary buds (AXBs) with distinguishable anatomical features such as leaf primordia. In literature, the terms AXM and AXB are sometimes used interchangeably, causing confusion. In this work, we use the term AXB to describe a macroscopically visible but visually undifferentiated organ located in the leaf axil. AXBs can either grow out as vegetative or generative branches or remain dormant, depending on the SAM status (reviewed in Leyser, 2003). Dormancy was originally defined as “lack of visible growth in a meristematic tissue” (Lang, 1987), and in this work, we apply this definition to AXBs. In the economically important Rosaceae family, AXBs can either remain dormant or form a short shoot with short internodes and leaf rosette-like appearance. The third fate is to form long shoots characterized by long internodes. Because different shoot types have different capabilities for forming inflorescence meristems and have different growth vigor, AXB fate has major consequences on plant architecture, yield potential and environmental adaptation of the plant (Costes et al., 2014). In strawberry, AXB fate directly affects yield potential, as inflorescences are formed at the apices of short shoots called branch crowns (BCs) (Battey et al., 1998). Long shoots of strawberry, known as stolons or runners, are indispensable for clonal propagation of the crop. Although a handful of genes regulating AXB fate in strawberry has been identified, environmental regulation of AXB fate in this important crop remains obscure.

In Arabidopsis, AXB outgrowth depends on both endogenous and environmental factors. The most important endogenous factor is apical dominance exerted by the SAM controlling lateral bud outgrowth (Cline, 1997). Apical dominance functions, at least in part, via auxin produced by the SAM. Auxin travels downward through the phloem, promoting the synthesis of strigolactones while inhibiting the activity of cytokinins. Unlike auxin, these hormones can move into AXBs to inhibit and promote bud outgrowth, respectively (Skoog and Thimann, 1934; Dun et al., 2012; Rameau et al., 2015; Qiu et al., 2019). Both strigolactones and cytokinins have been shown to regulate the expression of *BRC1* (*BRANCHED1*; Dun et al., 2012), a TCP (TEOSINTE BRANCHED1, CYCLOIDEA, PCF) transcription factor that is expressed in growth-arrested AXBs both in monocots and dicots (Takeda et al., 2003; Aguilar-Martinez et al., 2007; Braun et al., 2012).

The above-mentioned mechanisms for controlling AXB fate have been elucidated in annual species. A genetic framework for controlling AXB fate in perennial species has started to emerge only recently, mainly based on results obtained in hybrid aspen (*Populus tremula* × *Populus tremuloides*). Specific components of the regulatory network controlling bud outgrowth are conserved between hybrid aspen and annual plants; the putative tree orthologs of *BRC1* inhibit bud outgrowth (Muhr et al., 2018) and are regulated by strigolactones (Muhr et al., 2016). However, aspen *BRC1* is also regulated by a negative feedback loop involving putative tree orthologs of *TERMINAL FLOWER1* (*TFL1*) and *APETALA1* (*LIKE-APETALA1*/*LAP1*) (Maurya et al., 2020). Aspen *TFL1* represses *LAP1* expression, which in turn is a negative regulator of both *TFL1 and BRC1*. SDs (short days) and low temperature upregulate *TFL1*, leading to downregulation of *LAP1* and de-repression of *BRC1*. Upregulation of *BRC1* by conditions mimicking oncoming winter inhibits bud outgrowth and suppresses branching (Maurya et al., 2020).

The role of gibberellins in the outgrowth of AXBs in hybrid aspen was recently suggested by Katyayini et al. (2020). They found that different bioactive GA forms have opposing roles in regulating AXB outgrowth as a response to shoot decapitation. In growth-arrested AXBs of hybrid aspen, GA3 and GA6 upregulate catabolic *GA2ox* genes, whose protein products deactivate GA1 and GA4. Thus, AXB quiescence is maintained despite active ongoing GA biosynthesis and is released upon loss of apical dominance by strong downregulation of *GA2ox* genes (Katyayini et al., 2020). However, care should be taken when interpreting these results because they are based on exogenous hormonal applications.

In both woodland strawberry (*Fragaria vesca*) and cultivated strawberry (*F*. × *ananassa*), AXB fate is controlled by environmental and genetic factors. In general, flowering inducing conditions promote the formation of BCs analogous to short spur shoots in Rosaceous fruit trees. Strawberry inflorescences arise terminally at the SAM of the main crown and the uppermost AXB(s) of the main crown form BC(s), thus following a sympodial growth pattern (Darrow, 1966; Guttridge, 1985). Under nonflowering-inducing conditions, AXBs may remain dormant in a visually undifferentiated state or produce stolons, a form of asexual reproduction (Brown and Wareing, 1965; Darrow, 1966; Hytönen et al., 2009). It remains unknown whether the environmental conditions favoring floral induction regulate the AXB fate directly, or whether the floral induction at the SAM induces BC development indirectly, by e.g. releasing AXBs from growth arrest caused by apical dominance.

Strawberry genotypes are classified based on their photoperiodic flowering responses into either seasonal or perpetual flowering types. In seasonal flowering *F. vesca* genotypes, SDs and cool temperatures promote flowering and AXB development into BCs, while LDs (long days) and warm temperatures enhance stolon development. In contrast, perpetual flowering *F. vesca* genotypes initiate inflorescences and develop BCs in LD conditions, while SDs and warm temperature promote stolon development (Brown and Wareing, 1965; Mouhu et al., 2009; Sønsteby and Heide, 2008). In seasonal flowering *F*. × *ananassa* cultivars, the environmental responses are similar to those of seasonal *F. vesca*. Seasonal flowering *F*. × *ananassa* cultivars are induced to flower and develop BCs under SDs, and their stolon development is promoted by LDs and warm temperature (Heide, 1977). In perpetual flowering *F*. × *ananassa* cultivars, the environmental responses are more variable than in perpetual flowering *F. vesca* genotypes. For example, SDs and high temperature (27°C) strongly promoted stolon development in the perpetual flowering F1 hybrid “Elan” (Sønsteby and Heide, 2007a, 2007b). However, LDs at 27°C promoted stolon development in the perpetual flowering cultivars ‘Rita’, ‘Ridder’, and ‘Flamenco’, whereas the cultivar ‘Rondo’ appeared to develop stolons independently of photoperiod (Sønsteby and Heide, 2007b).

Several studies have shown an association between AXB fate and GA in the two strawberry species. The early study by Thompson and Guttridge (1959) demonstrated that GA application to a SD-grown seasonal flowering *F*. × *ananassa* cultivar can mimic the effect of LD conditions; GA-treated plants developed stolons instead of BCs and did not initiate flowers in contrast to nontreated control plants. This finding was later expanded by Hytönen et al. (2009), who showed that AXB development into stolons in *F*. × *ananassa* requires bioactive GA1 and that inhibiting GA biosynthesis by prohexadione-calcium (Pro-Ca, an inhibitor of GA biosynthesis) increases the proportion of AXBs developing into BCs. Recently, the requirement for bioactive GA for stolon development in *F. vesca* was suggested by Tenreira et al. (2017), who provided evidence that the stolonless phenotype observed in certain perpetual flowering *F. vesca* accessions is caused by a loss-of-function mutation in *FvGA20ox4*. GA20-oxidases are GA biosynthetic enzymes that produce precursors of bioactive GA that are then converted to bioactive GA1 and GA4 by GA3-oxidases. Further down in the pathway, GA2-oxidases control the pool of bioactive GA by catalyzing their inactivation (Hedden, 2020). The importance of GA signaling downstream of the GA biosynthetic pathway was demonstrated by Caruana et al. (2018), who showed that the stolonless *F. vesca* phenotype can be reversed by a loss of function mutation in *FveRGA1*, encoding a DELLA growth repressor in GA signaling pathway (Peng et al., 1997). Due to the mutation, the GA signaling pathway is constitutively switched on, resulting in continuous stolon development in the normally stolonless perpetual flowering *F. vesca* accession (Caruana et al., 2018). A recent study by (Feng et al., 2021) described a mutant of *loss of axillary meristems* (*lam*), defective in AXM initiation. However, the *lam* mutation did not affect AXB fate in the tested *F*. *vesca* genotypes, nor was the effect of the mutation tested under different environmental conditions.

Although the importance of the GA pathway for determining AXB fate in strawberry has been demonstrated, the upstream regulation of the GA pathway remains a less-studied topic, especially in terms of environmental regulation. However, it has been shown that the *F. vesca* MADS box transcription factor FvSOC1 relays photoperiodic signals to the GA pathway at 18°C and that altering *FvSOC1* expression either by overexpression or silencing obstructs the photoperiodic regulation of vegetative development in strawberry. Altered *FvSOC1* expression levels in *FvSOC1* transgenic plants changed the activity of several GA pathway genes in leaf tissues (Mouhu et al., 2013).

*FvSOC1* is photoperiodically regulated via the FvCO–FvFT1 (*F. vesca* CONSTANS1; *F. vesca* FLOWERING LOCUST1) pathway that is active exclusively under LD conditions (Rantanen et al., 2014; Kurokura et al., 2017). FvSOC1 relays photoperiodic signals not only to the GA pathway, but also to the photoperiodic flowering pathway by upregulating *FvTFL1*, a strong floral repressor (Koskela et al., 2012; Mouhu et al., 2013). In seasonal flowering accessions, activity of the FvFT1–FvSOC1–FvTFL1 pathway represses flowering in LDs. Perpetual flowering accessions carry a mutation in the *FvTFL1* coding sequence that inhibits its function as a floral repressor. Since the flowering decision depends on the balance between FvFT1 and functional FvTFL1, the abundance of FvFT1 under LD conditions coupled to the presence of nonfunctional FvTFL1 enhances flowering in perpetual flowering accessions (Koskela et al., 2012; Mouhu et al., 2013; Rantanen et al., 2015)

A complicating factor in studying vegetative responses in strawberries is the dependence of AXB fate on the SAM state. Once the SAM is committed to forming an inflorescence meristem, apical dominance is released and the uppermost AXBs of the main crown develop into BCs (Darrow, 1966; Guttridge 1985, Sugiyama et al., 2004). However, the SAM fate cannot be the only factor regulating AXB fate, because AXBs do develop into BCs photoperiod-dependently also in genotypes where the SAM is forced to remain in vegetative state by overexpressing *FvTFL1* (Koskela et al., 2012).

In this article, we study the environmental regulation of AXB fate independently of that of the SAM by using a number of natural *F. vesca* accessions and transgenic genotypes with different environmental responses and flowering characteristics. Our analyses on *FvGA20ox4* transgenic plants also confirm that *FvGA20ox4* is indispensable for stolon development and show that the activity of *FvGA20ox4* is tightly regulated by both photoperiod and temperature. Finally, we show that *FvGA20ox4* is photoperiodically regulated via FvSOC1 at 18°C and provide evidence for an *FvSOC1*-independent pathway controlling vegetative development at 22°C.

## Results

### *FvGA20ox4* expression is required for stolon development

**Figure 1:**
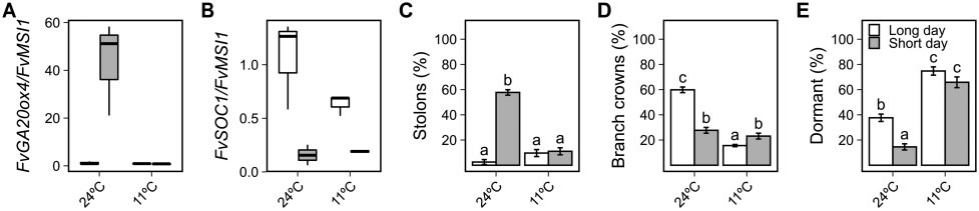
Stolon development is environmentally regulated via the activation of *FvGA20ox4* in perpetual flowering woodland strawberry. Relative expression of *FvGA20ox4* (A) and *FvSOC1* (B). Percentage of axillary buds developing stolons (C), or BCs (D), or remaining dormant (E). WT Hawaii-4 seedlings (*n* = 13–20) were grown in growth chambers equipped with LED lamps under 18 or 12-h photoperiod at 24°C or 11°C for 10 weeks and axillary bud fates were recorded until the end of the experiment. For relative expression, three apices per each biological replicate (*n* = 3) were collected after 5 weeks. In (A and B), center lines represent the median and upper and lower hinges represent the first and third quartiles. Upper and lower whiskers extend from the upper hinge to 150% of the interquartile range and from the lower hinge to −150% of the interquartile distance, respectively. Points beyond the whiskers are outliers according to Tukey. In (C–E), error bars represent the standard error of the mean and different letters indicate significant differences calculated by logistic regression and Tukey’s test (*P* < 0.05).

Strawberry AXB can develop a stolon, a BC, or remain dormant (Darrow, 1966), and a recent study showed evidence, by using a natural stolonless *F. vesca* mutant grown under natural light conditions, that GA biosynthetic enzyme FvGA20ox4 was needed for stolon development (Tenreira et al., 2017). However, the role of *FvGA20ox4* in stolon development in *F. vesca* has not been confirmed by transgenesis, nor have the phenotypes of *FvGA20ox4*-deficient genotypes been examined under a controlled environment. To understand the function of *FvGA20ox4* in the control of AXB fate, we first explored photoperiodic and temperature regulation of *FvGA20ox4* mRNA expression and AXB fate in the perpetual flowering and stolon-forming wild-type (WT) genotype H4 (Mouhu et al., 2009; for the characteristics of the *F. vesca* genotypes used in this work, see Supplemental Table S1). By analyzing shoot apex samples containing young AXBs, we observed a much higher *FvGA20ox4* expression level in SDs at 24°C than in other temperature/photoperiod combinations (Figure 1). Because a previous study in seasonal flowering *F. vesca* showed that *FvSOC1* upregulates *FvGA20ox4* in leaves (Mouhu et al., 2013), we also analyzed *FvSOC1* expression. We found that LD conditions upregulated *FvSOC1* in shoot apices at both 24°C and 11°C (Figure 1), revealing a disconnection between *FvSOC1 and FvGA20ox4* at 24°C. Concordantly with *FvGA20ox4* expression, the majority of AXBs in plants grown under SDs at 24°C developed a stolon (Figure 1), showing that the higher *FvGA20ox4* mRNA expression level in SDs at 24°C is associated with AXB development into stolons in H4, while a higher percentage of AXBs developed into BCs in LDs (Figure 1). All plants grown under LDs at 24°C flowered after 7 weeks of the treatment, while all the plants grown under SDs or at 11°C remained vegetative, supporting previous findings that flower-inducing conditions promote BC development, and in vegetative stage, most AXBs develop into a stolon (Brown and Wareing, 1965; Kurokura et al., 2017). The cool temperature of 11°C promoted AXB dormancy independently of the photoperiod, while at 24°C AXB dormancy was promoted by LDs (Figure 1). Taking these data together, *FvGA20ox4* expression and thereby AXB fate are tightly controlled by both temperature and photoperiod in the perpetual flowering *F. vesca*, and this regulation mechanism is uncoupled from *FvSOC1* expression at 24°C

**Figure 2:**
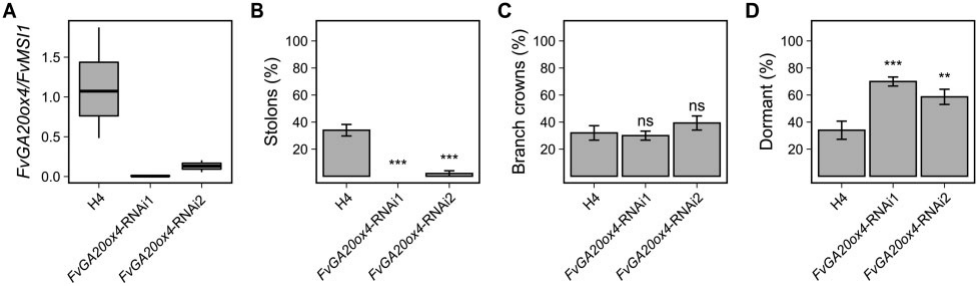
*FvGA20ox4* is required for stolon formation in perpetual flowering woodland strawberry. Relative *FvGA20ox4* expression (A). Percentage of axillary buds developing stolons (B), or BCs (C), or remaining dormant (D). WT Hawaii-4 and *FvGA20ox4*-RNAi seedlings (*n* = 10) were grown in a growth chamber equipped with LED tubes under 12-h photoperiod at 17°C. Axillary bud fates were recorded and apex samples collected when the plants had developed five fully opened leaves. For relative expression, six to eight apices per each biological replicate (*n* = 4) were collected. In (A), center lines represent the median and upper and lower hinges represent the first and third quartiles. Upper and lower whiskers extend from the upper hinge to 150% of the interquartile range and from the lower hinge to −150% of the interquartile distance, respectively. In (B–D), error bars represent the standard error of the mean and statistically significant differences, calculated by logistic regression and Dunnett’s test, are indicated by asterisks (***P < 0.0001; **P < 0.001; *P < 0.01); H4 = Hawaii-4.

To confirm the function of *FvGA20ox4* in controlling AXB fate, we transformed *FvGA20ox4*-RNAi construct into the perpetual flowering and stolon-forming H4 and obtained two transgenic lines with strong and gene-specific silencing of *FvGA20ox4* (Figure 2;Supplemental Figure S1). We attempted to force stolon development in these plants by growing them in SDs at 17°C and found that one of the *FvGA20ox4*-RNAi lines completely lacked stolons, and another line with weaker silencing of *FvGA20ox4* rarely produced stolons (Figure 2). In WT H4, in contrast, ∼80% of the AXBs developed into stolons. The *FvGA20ox4*-RNAi lines developed BCs at the same frequency as the WT (Figure 2), suggesting that silencing of *FvGA20ox4* had a specific effect on stolon development without interfering with BC development. However, the AXBs that were inhibited from developing a stolon due to silencing of *FvGA20ox4* remained dormant, resulting in a higher proportion of dormant AXBs in RNAi lines compared with WT (Figure 2). These data indicate that the absence of *FvGA20ox4* expression in AXBs does not directly lead to BC development, nor does it inhibit it. All WT and RNAi plants remained vegetative until the end of the experiment confirming that the silencing of *FvGA20ox4* affected AXB fate directly and not indirectly through floral development at SAM. Taken together, our results on RNAi lines demonstrated that *FvGA20ox4* is needed for stolon development and that additional factor(s) are required for BC development in the absence of *FvGA20ox4* expression.

### *FvGA20ox4* activity releases axillary bud dormancy

**Figure 3:**
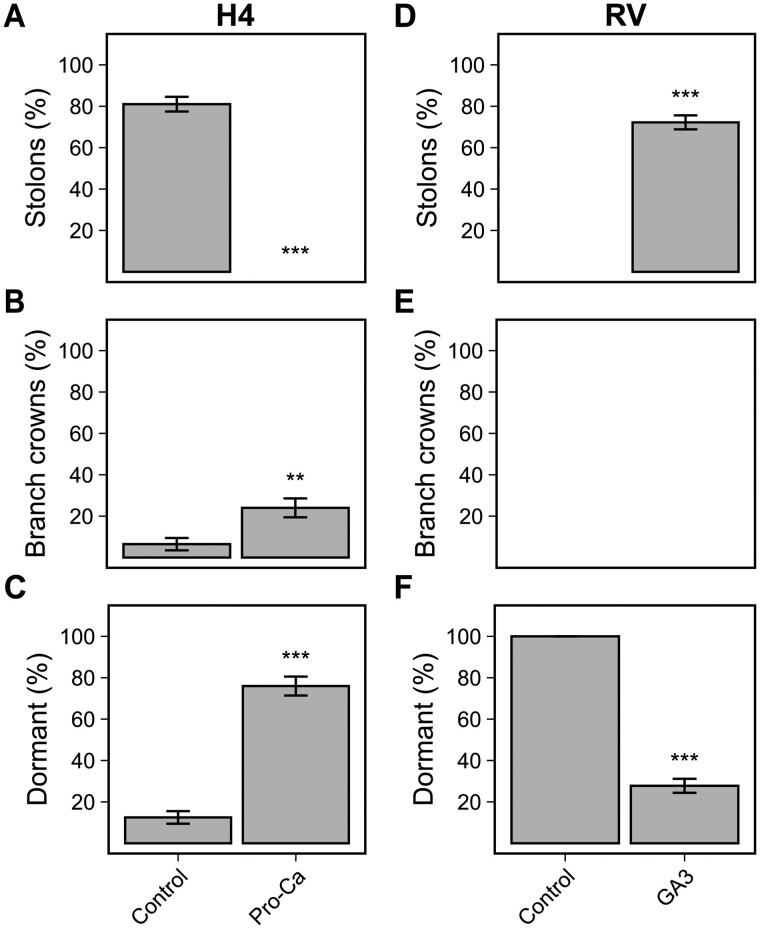
Gibberellic acid is required to release axillary bud dormancy in perpetual flowering woodland strawberry. Percentage of axillary buds developing stolons (A and D), BCs (B and E), or remaining dormant (C and F). In (A–C), WT Hawaii-4 seedlings (*n* = 17) were grown in a growth chamber equipped with LED lamps under 12-h photoperiod at 24°C and sprayed with Pro-Ca. In (D–F), RV seedlings (*n* = 35) were grown under 12-h photoperiod at 24°C and sprayed with GA3. In both experiments, the seedlings were sprayed when they had two to three fully opened leaves and axillary bud fates were recorded 2 weeks later when the plants had developed five to six leaves. Only the axillary buds developed after the treatment were taken into account. Error bars represent the standard error of the mean and statistically significant differences, calculated by logistic regression and Dunnett’s test, are indicated by asterisks (***P < 0.0001; **P < 0.001; *P < 0.01); H4 = Hawaii-4.

Since the silencing of *FvGA20ox4* increased the proportion of dormant AXBs in perpetual flowering and stolon-forming H4 (Figure 2), we carried out growth regulator treatments to further test the connection between GA and AXB dormancy. First, we sprayed young H4 seedlings with Pro-Ca, a GA biosynthesis inhibitor (Evans et al., 1999), and observed AXB fates in SD conditions to maintain the plants in vegetative phase. While untreated plants profusely produced stolons, Pro-Ca-treated plants did not develop any stolons (Figure 3). BCs were rarely observed in control plants during the 2 weeks of observations in this experiment, and Pro-Ca treatment only slightly although significantly increased the proportion of AXBs developing into BCs (Figure 3). The majority of AXBs remained dormant after the Pro-Ca treatment supporting the hypothesis that GA is required to break AXB dormancy in *F. vesca* in vegetative phase (Figure 3).

To test this hypothesis from different angle, we carried out GA3 treatment on seedlings of stolonless perpetual flowering genotype ‘Reine des Vallées’ (RV) (Supplemental Figure S2) grown in SDs. The GA3 treatment strongly promoted AXB outgrowth as stolons in vegetative seedlings, whereas no AXB outgrowth was observed in control plants during the first 2 weeks in SD (Figure 3). Also, a transfer of nontreated RV plants from SDs to flower-inductive LDs rapidly released AXB dormancy, but in these conditions, AXBs developed into BCs (Supplemental Figures S2 and S3). In conclusion, our growth regulator experiments and studies in *FvGA20ox4* RNAi lines revealed that both active GA biosynthesis in the AXB and application of synthetic GA can overcome AXB dormancy in *F. vesca* . This effect has not been observed in previous studies that used flower-induced plants (Tenreira et al., 2017).

**Figure 4:**
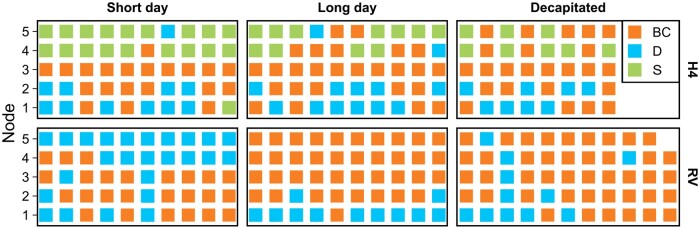
Decapitation releases axillary buds from apical dominance and leads to BC development. Short day-grown seedlings of perpetual flowering Hawaii-4 and RV were either decapitated at 4–6 leaf stage, left intact and maintained under short-day conditions, or moved to long-day conditions. The plants were grown at 24°C in growth chambers equipped with LED tubes. Axillary bud fates were scored weekly until nearly all available axillary buds in the decapitated seedlings had differentiated. Each square depicts the fate of an individual axillary bud; H4 = Hawaii-4, D = dormant, S = stolon.

AXB dormancy may be caused by apical dominance exerted by the actively growing vegetative SAM or by environmental conditions. AXBs can be released from apical dominance by decapitation (Cline, 1997). To dissect the effect of apical dominance from the environmental effect on AXB dormancy, we followed AXB fates in perpetual flowering H4 and RV plants grown at 24°C under three different treatments: SDs, LDs, and decapitation in SDs. In the stolonless RV, both LD conditions and decapitation of SD-grown plants promoted the development of the youngest AXBs into BCs, whereas intact SD-grown plants had a higher proportion of dormant AXBs (Figure 4;Supplemental Table S2). Also in the stolon-forming H4, LDs and decapitation in SDs promoted BC development, although the effect was less clear due to profuse stolon development (Figure 4;Supplemental Table S2). The fates of the older AXBs were not affected by decapitation or LD in either accession. These data demonstrate that the removal of apical dominance by decapitation is sufficient to release dormancy of the 1–2 youngest AXBs and to induce BC development under SD conditions in perpetual flowering *F. vesca* accessions. On the other hand, decapitation does not increase the proportion of AXBs developing stolons in H4. Taken together, FvGA20ox4 promotes stolon development irrespectively of apical dominance, whereas BC development from the youngest AXBs is inhibited by apical dominance.

### Direct environmental control of *FvGA20ox4* expression and AXB fate in the seasonal flowering woodland strawberry

We have so far confirmed that the expression of *FvGA20ox4* is indispensable for SD- and warm temperature-promoted stolon development in perpetual flowering *F. vesca* accessions. As opposed to perpetual flowering accessions, stolon development in seasonal flowering *F. vesca* genotypes is promoted by LD conditions and high temperature (Heide and Sønsteby, 2007; Mouhu et al., 2013). Because of the opposing photoperiodic responses, we wanted to gain evidence of the role of *FvGA20ox4* in the control of AXB fate in seasonal flowering *F. vesca* . First, we assessed the expression of all four *FvGA20ox* genes present in the latest *F. vesca* genome annotation (v.4.0.2; Li et al., 2019; Supplemental Table S3). Only *FvGA20ox2 and FvGA20ox4* expression was detected in the SAMs and AXBs collected from plants grown under SDs or LDs at 18°C (Supplemental Figure S4). In AXBs collected from LD-grown plants, the mRNA level of *FvGA20ox4* was more than one thousand times higher than that of *FvGA20ox2* (Supplemental Figure S4), suggesting that *FvGA20ox4* encodes a major GA20ox enzyme catalyzing the biosynthesis of the precursors of bioactive GA in AXB. Moreover, the expression of *FvGA20ox4* was more than approximately 100 times higher in AXBs compared with shoot apex samples (Supplemental Figure S4). According to these data, gibberellin biosynthesis in the AXBs of seasonal and perpetual flowering *F. vesca* accessions is dependent on the activity of the same GA20ox enzyme, encoded by the *FvGA20ox4* gene.

**Figure 5:**
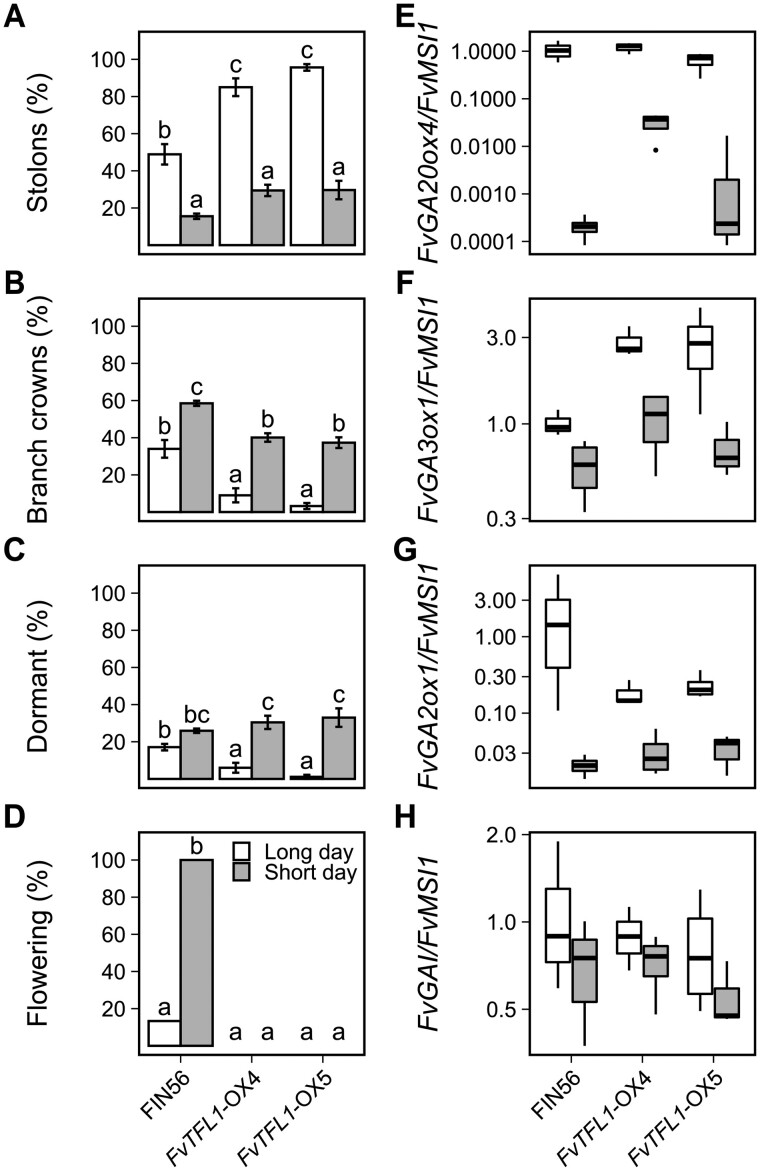
Photoperiod controls axillary bud fate at 18°C independently of shoot apical meristem fate. Percentage of axillary buds developing stolons (A), BCs (B), or remaining dormant (C). Percentage of flowering plants (D). Relative expression of *FvGA20ox4* (E), *FvGA3ox1* (F), *FvGA2ox1* (G), and *FvGAI* (H) in axillary bud samples. Stolon-propagated plants from *FvTFL1*-OX lines #4 and #5 in FIN56 background, and WT FIN56 were grown in a greenhouse under 18 or 12-h photoperiod at 18°C for 6 weeks. Axillary bud observations were recorded up to week 4 (*n* = 10–15) and flowering was scored up to Week 10. In (A–D), error bars represent the standard error of the mean and different letters indicate significant differences calculated by logistic regression and Tukey’s test (*P* < 0.05). For relative expression, axillary bud samples including five axillary buds per each biological replicate (*n* = 4) were collected on week 4. In (E–H), center lines represent the median and upper and lower hinges represent the first and third quartiles. Upper and lower whiskers extend from the upper hinge to 150% of the interquartile range and from the lower hinge to −150% of the interquartile distance, respectively. Points beyond the whiskers are outliers according to Tukey.

Next, we wanted to study how the photoperiod affects AXB fate in the seasonal flowering *F. vesca*, and whether this effect is direct or dependent on the SAM fate. If the fates of the AXBs were uncoupled from the fate of the SAM, SDs should inhibit stolon development and promote BC development even if the SAM remains vegetative. To test this hypothesis, we compared photoperiodic responses of seasonal flowering FIN56 accession with genotypes that remain vegetative in SDs at 18°C including *FvTFL1*-overexpression lines in FIN56 background (Koskela et al., 2012) and a natural vernalization-requiring accession NOR1 which shows abnormally high *FvTFL1* expression in SDs (Koskela et al., 2017). Photoperiod had a highly significant impact on AXB fate, triggering similar responses in FIN56, *FvTFL1*-overexpression lines, and NOR1. In all genotypes, more stolons developed in LDs compared with SDs, while SDs enhanced BC formation and increased the proportion of dormant buds (Figure 5;Supplemental Figure S5). However, the effect of SDs on BC development was more pronounced in FIN56 than in the other genotypes. Flowering was observed only in FIN56; all FIN56 plants flowered in SDs, and in LDs only two plants (13.3%) flowered. In summary, stolon development was inhibited by SDs independently of the SAM fate, whereas BC development depended both on the photoperiod and on the SAM status.

We also explored the effect of photoperiod on the mRNA expression of *FvGA20ox4* and other genes of the GA biosynthetic, catabolism, and signaling pathways in seasonal flowering FIN56 and *FvTFL1* overexpression lines. In consistence with the role of *FvGA20ox4* in the control of AXB fate in the perpetual flowering woodland strawberry (Figure 1), we found strong downregulation of *FvGA20ox4* mRNA levels in SDs compared with LDs in both FIN56 and the transgenic lines (Figure 5) confirming the direct photoperiodic regulation of this gene in AXBs. Among the other GA pathway genes studied by Mouhu et al. (2013) in leaf tissues, *FvGA3ox1*, *FvGA2ox1*, and *FvGAI* (*F. vesca GIBBERELLIC ACID INSENSITIVE*) exhibited high expression levels in AXB samples (Figure 5). Both *FvGA3ox1 and FvGA2ox1* were clearly downregulated in SDs in both FIN56 and *FvTFL1* overexpression lines (Figure 5). However, no major differences between photoperiods were found in the expression of *FvGAI* (Figure 5;Supplemental Table S3), which encodes a major DELLA protein in *F. vesca* (Caruana et al., 2018; Li et al., 2018). These data demonstrate that the GA biosynthetic pathway in AXBs of seasonal flowering *F. vesca* is regulated directly by photoperiod and does not depend on floral induction in the SAM.

**Figure 6:**
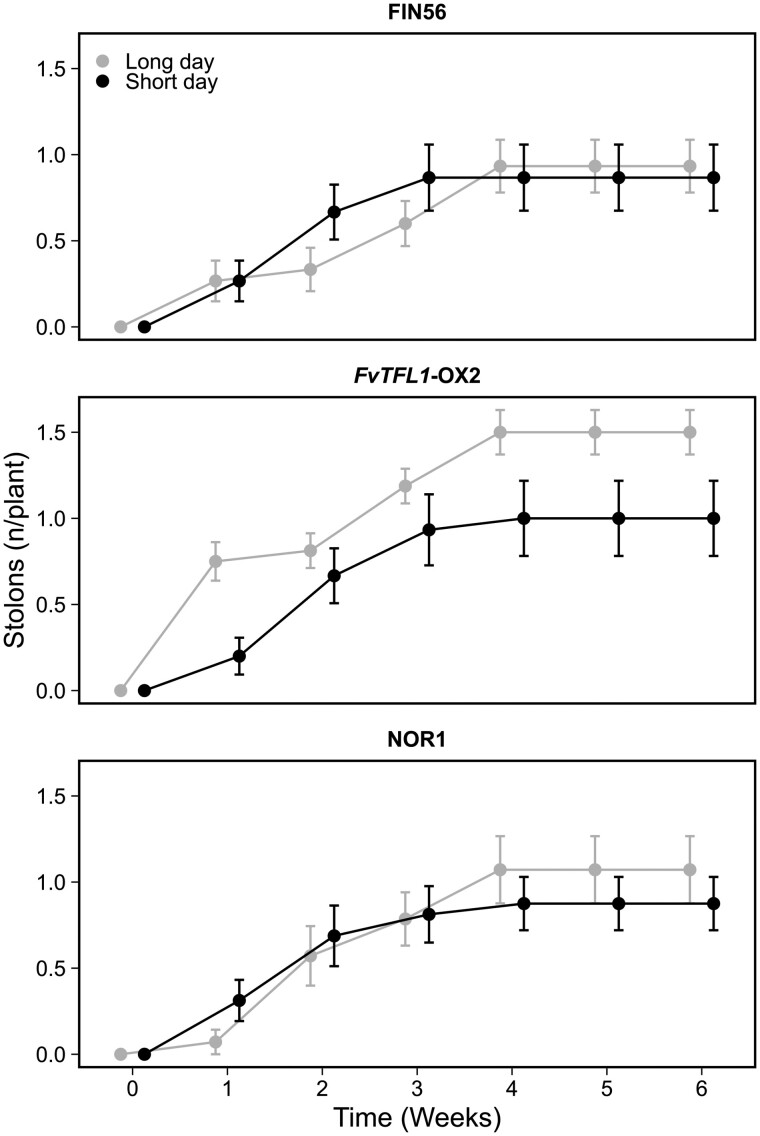
Cool temperature stops stolon development. Stolon-propagated plants of seasonal flowering wild type FIN56, *FvTFL1-*OX line #2 in FIN56 background, and NOR1 were subjected to photoperiod treatments (18 or 12-h daylight) at 10°C for 5 weeks. Plants were grown in growth chambers equipped with LED lamps during the treatments and then transferred to a greenhouse under long days at 18°C. Axillary bud observations were recorded up to Week 6. Error bars represent standard error (*n* = 14–16).

So far, we concentrated on the role of photoperiod in the regulation of AXB fate. To understand the role of temperature in the control of AXB fate in more detail, we explored the responses of seasonal flowering FIN56, one *FvTFL1* overexpression line, and NOR1 to 10°C temperature in SDs and LDs. Stolon development ceased after 4 weeks in all genotypes under both photoperiods (Figure 6), and a relatively high proportion of AXBs developed into BCs in FIN56 and NOR1 (Supplemental Figure S6). In FIN56, almost all plants flowered in both photoperiods, but in other genotypes, only a single NOR1 plant flowered (Supplemental Table S4), showing that 10°C affected AXB fate independently of the SAM developmental status or photoperiod in NOR1 and *FvTFL1* overexpression plants.

**Figure 7:**
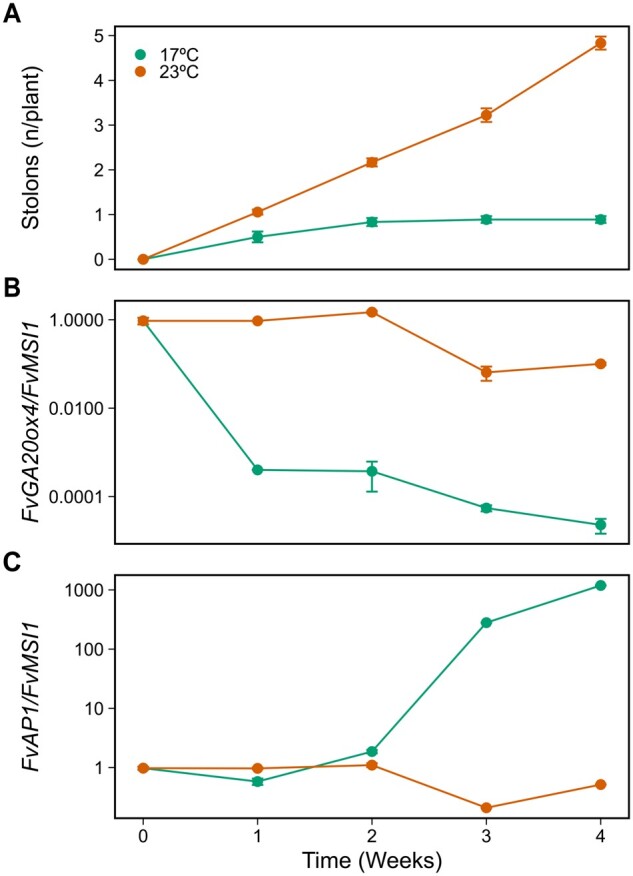
*FvGA20ox4* is environmentally regulated in seasonal flowering FIN56. The number of stolons developed per plant (A), relative *FvGA20ox4* expression in axillary bud samples (B), and relative *FvAP1* expression in apex samples (C). FIN56 plants (*n* = 18) were grown under 12-h daylight at 17°C and 23°C for 4 weeks and axillary bud fates were recorded weekly. For relative expression, samples including three axillary buds (B) or three apices (C) per each biological replicate (*n* = 3) were collected weekly. Error bars represent the standard error of the mean.

Next, we carried out time-course expression analyses in AXB samples in parallel with stolon observations to explore how the temporal regulation of *FvGA20ox4* associates with AXB fate in seasonal flowering FIN56 transferred from LD conditions to SDs at 17°C or 23°C. In SDs at 17°C, stolon development ceased after 14 d (Figure 7). This was associated with an almost complete shutdown of *FvGA20ox4* mRNA expression already during the first week of the treatment, indicating that the strong photoperiodic regulation of *FvGA20ox4* controls AXB fate in FIN56 at 17°C (Figure 7). However, in SDs at 23°C *FvGA20ox4* expression level remained high and stolon development continued until the end of the experiment demonstrating a strong temperature effect on the expression of *FvGA20ox4* and AXB fate.

To explore the connection of these AXB observations with the developmental status of the SAM, we also analyzed the expression of the floral marker gene *Fragaria vesca APETALA1 (FvAP1*; Koskela et al., 2012) in SAM samples. In SDs at 17°C, *FvAP1* mRNA level remained low until Day 14, but increased abruptly thereafter indicating that flower induction happened between Days 14 and 21, while the lack of the *FvAP1* activation at 23°C showed that plants remained in vegetative phase at this temperature (Figure 7). In all the analyses above, independently of the photoperiodic response type of the plants (perpetual or seasonal flowering), stolon development was associated with the vegetative status of the SAM, which is in line with previous studies (Mouhu et al., 2013; Kurokura et al., 2017; Tenreira et al., 2017). However, our results show that the downregulation of *FvGA20ox4* in AXBs and the cessation of stolon development occur before the upregulation of *FvAP1* in the SAM of SD-grown FIN56 suggesting temporal separation in the environmental control of AXB and SAM fates.

### The role of *FvSOC1* in the regulation of AXB fate

**Figure 8:**
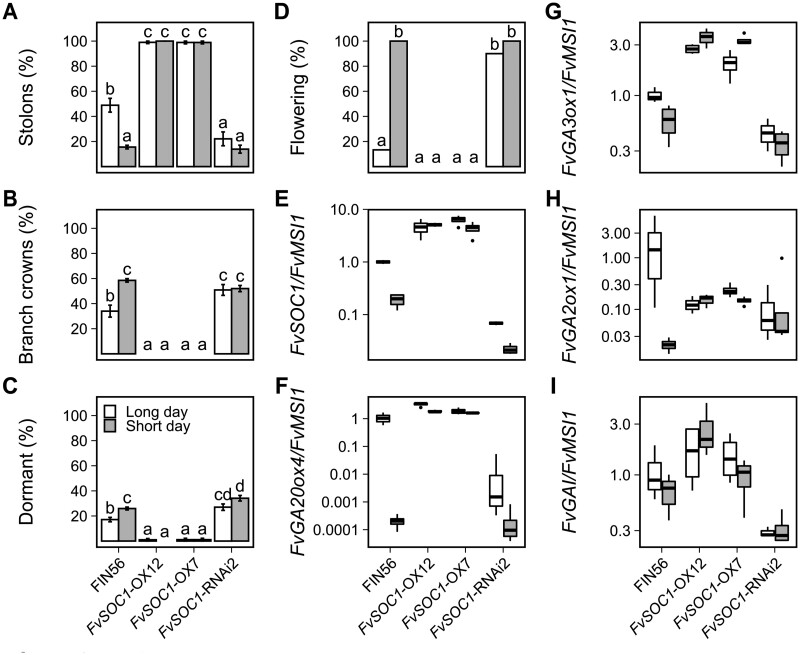
*FvSOC1* regulates gibberellic acid biosynthesis in axillary buds at 18°C. Percentage of axillary buds developing stolons (A), BCs (B), or remaining dormant (C). Percentage of flowering plants (D). Relative expression of *FvSOC1* (E), *FvGA20ox4* (F), *FvGA3ox1* (G), *FvGA2ox1* (H), and *FvGAI* (I) in axillary bud samples. Stolon-propagated plants (*n* = 10 − 15) of seasonal flowering WT FIN56, *FvSOC1–*OX7, *FvSOC1–*OX12, and *FvSOC1–*RNAi2 in FIN56 background were grown in the greenhouse under 18 or 12-h photoperiod at 18°C for 6 weeks. Axillary bud observations were recorded up to Week 4 and flowering was scored up to Week 10. In (A–D), error bars represent the standard error of the mean and different letters indicate significant differences calculated by logistic regression and Tukey’s test (*P* < 0.05). For relative expression, axillary bud samples including five axillary buds per each biological replicate (*n* = 4) were collected on week 4. In (E–I), center lines represent the median and upper and lower hinges represent the first and third quartiles. Upper and lower whiskers extend from the upper hinge to 150% of the interquartile range and from the lower hinge to −150% of the interquartile distance, respectively. Points beyond the whiskers are outliers according to Tukey.

To investigate the genetic regulation of AXB fate upstream of the GA pathway, we focused on *FvSOC1*. We reanalyzed photoperiodic responses of *FvSOC1* overexpression and silenced lines generated by Mouhu et al. (2013) and explored the expression of GA pathway genes in their AXBs. In consistence with previous results (Mouhu et al., 2013), we found that both *FvSOC1* overexpression and silenced lines had lost the normal photoperiodic control of AXB fate and flowering at 18°C. In the overexpression lines, almost all AXBs developed a stolon in both photoperiods, and none of the plants flowered (Figure 8). FIN56 developed stolons from about half of the AXBs in LDs, while the *FvSOC1*-RNAi silenced line developed significantly less stolons in these conditions. However, SDs reduced the proportion of AXBs developing a stolon in FIN56, but not in the silenced line. Furthermore, the silenced line produced slightly more BCs under LDs than FIN56, and more AXBs remained dormant in silenced line in both photoperiods. All FIN56 plants flowered in SDs, while the *FvSOC1* silenced line flowered in both photoperiods. These data demonstrate that *FvSOC1* is required for relaying information on photoperiod to the AXBs, and that artificially increasing *FvSOC1* abolishes both photoperiodic and temperature regulation of AXB fate.

Next, we analyzed the expression of *FvSOC1* and GA pathway genes in AXBs of seasonal flowering FIN56 plants grown at 18°C. *FvSOC1* mRNA level was clearly higher in *FvSOC1* overexpression lines and lower in the silenced line than in FIN56, and clear downregulation of this gene was found in SDs compared with LDs in both FIN56 and the silencing line (Figure 8). The expression of *FvGA20ox4* and *FvGA3ox1* followed the patterns of *FvSOC1* expression in both transgenic lines and FIN56 grown in different photoperiods (Figure 8) supporting the role of FvSOC1 in the activation of GA biosynthetic genes in AXBs. The *FvGA2ox1* gene encoding a GA catabolic enzyme followed the same pattern of photoperiodic regulation as the biosynthetic genes in the WT FIN56, with LDs upregulating expression (Figure 8). However, in the *FvSOC1* overexpression and silenced lines, the photoperiodic regulation of *FvGA2ox1* was clearly abolished. Furthermore, lower expression level of *FvGAI* was detected in *FvSOC1* silenced line compared with FIN56 (Figure 8), and the overexpression lines had slightly higher levels of *FvGAI* mRNA than the wild type.

Once we revealed the key role of *FvSOC1* in the photoperiodic regulation of AXB fate at 18°C, we tested its function at different temperatures by growing WT FIN56, *FvSOC1*-OX and RNAi plants at 10°C and 22°C in both LDs and SDs and observed AXB fates. Due to lack of plant material, the *FvSOC1* silenced line (*FvSOC1*–RNAi3) we used in this experiment was not tested at 18°C temperature in the experiments described in this article, but the line was earlier shown to develop less stolons than the WT FIN56 when grown in LDs at 18°C (Mouhu et al., 2013). The transcript levels of *FvSOC1* in the *FvSOC1*–RNAi lines grown at 23°C have been previously characterized by Rantanen et al. (2015), who showed that *FvSOC1* is silenced in these lines at 23°C. In FIN56, 10°C inhibited and 22°C promoted stolon development independently of the photoperiod (Supplemental Figure S7). The *FvSOC1*-OX plants developed stolons profusely, independently of the environmental conditions. In contrast to the results presented here at 18°C (Figure 8) and in Mouhu et al. (2013), the *FvSOC1*–RNAi3 plants grown in LDs at 22°C developed stolons at the same frequency as the WT FIN56 (Supplemental Figure S7). This means that in FIN56, *FvSOC1* is not required for stolon development at 22°C, and that an *FvSOC1*-independent pathway controls AXB fate at warm temperature in the seasonal flowering woodland strawberry.

## Discussion

Studies in *F. vesca* on the effect of photoperiod and temperature have previously focused on flowering responses, while data on how the environment affects vegetative growth remain scarce. Moreover, previous studies (Sugiyama et al., 2004; Tenreira et al., 2017) have speculated that the AXB fate depends on the flowering decision made at the SAM. In other words, floral-inducing environmental conditions would have an indirect effect on AXB fate because floral induction at SAM forces BC development from AXBs. Here, we use functional genetics, growth regulator treatments, and controlled-climate experiments to demonstrate that environmental conditions affect the activity of the GA biosynthetic pathway and AXB fate directly, and show that the fate of the SAM affects only on the fates of the youngest AXBs. We also suggest a role for *FvGA20ox4* in releasing strawberry AXBs from growth arrest caused by apical dominance and provide evidence for an *FvSOC1*-independent high-temperature activated pathway promoting stolon development.

### *FvGA2o0x4* activity overrides the effect of apical dominance and promotes stolon development

Previous studies on the role of GA in strawberry vegetative responses have suggested that the presence of bioactive GA in the AXBs leads to stolon development, while in the absence of GA BCs are formed (Hytönen et al., 2009; Tenreira et al., 2017; Feng et al., 2021). However, these studies have either used flower-induced plants (Tenreira et al., 2017; Feng et al., 2021), or have not observed the frequency of dormant AXBs on the main crown (Hytönen et al., 2009). Our current results are based on careful observations of AXB fates of the main crown of both flower-induced and noninduced plants and provide evidence for a fine-tuned regulatory mechanism that involves the *FvGA20ox4* as an on/off switch controlling stolon development but also suggests the presence of an active mechanism controlling BC development and AXB dormancy.

In consistence with a number of earlier studies that showed GA as a factor promoting stolon development (e.g. Thompson and Guttridge, 1959; Hytönen et al, 2009; Mouhu et al., 2013; Tenreira et al., 2017), our experiments revealed a clear connection between stolon development and the activity of *FvGA20ox4* in all examined woodland strawberry genotypes (Figures 1–3, 5, 7, and 8). In the perpetual flowering H4, stolon development was enhanced under SDs at warm temperatures. However, SD conditions failed to induce stolon development on *FvGA20ox4*–RNAi lines, confirming that *FvGA20ox4* is required for stolon development. BC development was not affected in the *FvGA20ox4*–RNAi plants (Figure 2), indicating that silencing *FvGA20ox4* had a specific effect on stolon development. The AXBs that were restrained from developing stolons due to silencing of *FvGA20ox4* remained dormant (Figure 2). We were able to reproduce the dormant-AXB phenotype in WTs of two LD-flowering accessions H4 and RV grown under nonflower-inductive SDs by treating the plants with growth regulators. It is noteworthy that the AXBs of vegetatively growing plants developed stolons in the presence of bioactive GA, but in the absence of GA, they remain dormant instead of developing into BCs (Figure 3). These results are at odds with the idea presented by Tenreira et al. (2017), who suggested that AXBs develop into BCs as a default setting whenever bioactive GA is absent.

### Axillary bud fate in the seasonal flowering strawberry is predominantly environmentally regulated

Earlier studies in the seasonal flowering *F. vesca* accession FIN56 have demonstrated that LDs promote stolon development and activate the expression of *FvGA20ox4* in leaf tissues (Mouhu et al., 2013). We show here for the first time that *FvGA20ox4* expression is activated by LDs in AXBs of FIN56 at 18°C, while at 23°C high expression level is found even in SDs (Figures 7 and 8, F). To summarize, *FvGA20ox4* activity associates with stolon development in all the tested genotypes and environmental conditions (Figures 5, 7, and 8). This is hardly a surprising finding, given that exogenous application of bioactive GA has been shown to promote stolon development in a number of studies (Thompson and Guttridge, 1959; Hytönen et al., 2009; Mouhu et al., 2013). Therefore, we wanted to go a step further and study how the environment affects AXB fate in general, and to dissect the indirect environmental effect via floral induction (in other words, the loss of apical dominance) from the direct environmental effect on AXB fate in the seasonal flowering *F. vesca*.

To achieve this, we studied the association between AXB fates and floral induction under different environmental conditions using genotypes with contrasting flowering characteristics. Stolon development is inhibited at 9°C or by SDs at 15°C, the conditions that induce flowering in seasonal flowering woodland strawberry genotypes (Heide and Sønsteby, 2007). Our data corroborate these results in FIN56 at 10°C (Figure 6) or SDs at 17–18°C (Figures 5 and 7), and also demonstrates that the inhibition of stolon development occurs independently of the SAM fate in *FvTFL1*-OX plants and NOR1 accession that remain vegetative under these conditions. Moreover, the inhibition of *FvGA20ox4* expression and stolon development in FIN56 by SDs occurs weeks before floral induction takes place (Figure 7), showing that these processes can be temporally separated.

We have confirmed that *FvGA20ox4* is indispensable for stolon development and it is environmentally regulated, but we do not know the identity of the gene(s) regulating BC development in *F. vesca*. A good starting point for such studies would be the analysis of AXB fate-related candidate genes identified by Samad et al. (2017). Their QTL analysis revealed an association between AXB fate and strawberry TCP transcription factor *FvTCP7* on LG4, as well as with two *DORMANCY-ASSOCIATED MADS* (*DAM*) *BOX* genes in LG5 (Samad et al., 2017). As *FvTCP7* belongs to the same transcription factor family as *BRC1*, a major regulator of bud outgrowth in *Arabidopsis* (Aguilar-Martinez et al., 2007), studying the function of *FvTCP7* could provide important insights into the control of BC formation in *Fragaria.* DAM genes have been associated with dormancy in a number of perennial species (reviewed in Falavigna et al., 2019). Functional studies of the LG5 *DAM* genes reported by Samad et al. (2017) are needed to confirm their roles in controlling AXB fate.

### *FvSOC1* is required for stolon development only at intermediate temperature

LDs have been shown to activate *FvSOC1*, leading to upregulation of *FvGA20ox4* in leaf tissues at 18° (Mouhu et al., 2013). Concordantly, in our photoperiodic experiments *FvSOC1* expression associated with the level of *FvGA20ox4 and FvGA3ox1* expression in AXBs in the seasonal flowering FIN56 plants grown at 18°C (Figure 8). Moreover, overexpressing or silencing *FvSOC1* abolished the normal photoperiodic responses (Figure 8), demonstrating that FvSOC1 is required for relaying photoperiodic information to regulate GA biosynthesis by transcriptional regulation of *FvGA20ox4* at 18°C. However, in seasonal flowering woodland strawberry, temperature of 22°C promoted stolon development in both the WT and *FvSOC1*-silenced lines independently of photoperiod (Supplemental Figure S7). In perpetual flowering *F. vesca*, *FvGA20ox4* expression and stolon development were activated under SDs at 24°C, although *FvSOC1* was expressed only weakly under these conditions (Figure 1). To summarize, our data provide evidence for an *FvSOC1*-independent and high temperature-activated pathway that promotes stolon development at temperatures over 22°C. This pathway shares an obvious analogy with the high temperature-activated and *FvSOC1*-independent pathway that upregulates *FvTFL1* and inhibits flowering at 23°C (Rantanen et al., 2015). These two pathways, one promoting *FvGA20ox4* expression and one upregulating *FvTFL1* at temperatures over 22°C, are likely to share regulatory components whose identities warrant further investigations.

### Regulation of axillary bud fate in strawberry

In this work, we aimed at elucidating regulation of AXB fate, and establishing whether AXB fate depends more on environmental or endogenous factors. Our data suggest that both types of factors are involved in the regulation AXB fate, and their effect depends on the position of the AXB on the plant.

The strongest effect is exerted by the SAM, but only on the youngest, uppermost AXBs. The fates of these buds depend directly on the fate of the SAM; if the SAM is induced to form an inflorescence, the uppermost AXBs form BCs, maintaining sympodial growth pattern. Our data on perpetual flowering *F. vesca* accessions (Figure 4) suggest that this effect is caused by apical dominance, whose effect can be overridden by the activity of *FvGA20ox4* to promote stolon development in the uppermost AXBs. Release from apical dominance, i.e. floral induction, appears to promote BC development also in the topmost AXBs of seasonal flowering *F. vesca*, as the SD-grown floral-induced FIN56 plants developed a significantly higher proportion of BCs than the nonflowering genotypes *FvTFL1*-OX and NOR1 (Figure 5). These data suggest that while environmental conditions are the major regulators of BC development in seasonal flowering *F. vesca*, floral induction directly regulates the fates of the youngest and uppermost AXBs.

In other sympodially growing species, such as cotton (*Gossypium hirsutum*) or tomato (*Lycopersicon esculentum*), the major determinants of the sympodial growth pattern are the *CETS* genes (Banfield and Brady, 2000), to which *TFL1* also belongs (Kobayashi et al., 1999). In both species, silencing the homologs of *TFL1* leads to AXB release from apical dominance and the abolishment of the sympodial growth habit (Pnueli et al., 2001; McGarry et al., 2016). In strawberry, the maintenance of apical dominance and the sympodial growth habit does not depend on *FvTFL1* activity, because the perpetual flowering accessions with nonfunctional mutated *FvTFL1* follow exactly the same sympodial growth mode (Brown and Wareing, 1965). Possible regulators of strawberry sympodial growth are the two *CENTRORADIALIS-LIKE* genes whose functions remain unexplored.

The second strongest effect on AXB fate is exerted by environmental conditions promoting stolon development. The environmental conditions that favor vegetative growth depend on the genotype; for perpetual flowering LD accessions, it is the combination of SDs and warm temperature (Figure 1), while in seasonal flowering accessions stolon development is promoted by LDs and/or warm temperature (Figures 5, 7, and 8; Hartmann, 1947; Konsin et al., 2001; Hytönen et al., 2004). Despite the contrasting environmental conditions, the mechanism for boosting stolon development is the same; GA biosynthetic pathway is activated in the vast majority of nondifferentiated AXBs, resulting in proliferative stolon development (Figures 1, 2, and 7). Abundant clonal reproduction has been shown to increase compensatory growth in case of damage to apical meristems in grasses (Liu et al., 2007). Therefore, the capability of strawberries of developing stolons is likely to be an important ecological adaptation that has contributed to the wide geographical distribution of strawberry species.

BC development from older AXBs is controlled by specific genotype-dependent environmental conditions (Figures 1, 2, and 5;Supplemental Figure S6). It is interesting that the environmental conditions promoting BC development also increase the proportion of dormant axillary buds (Figures 1, 2, and 5), both in perpetual flowering H4 and seasonal flowering FIN56 accessions as well as in *FvTFL1* overexpression lines in the FIN56 background. This phenomenon is likely to be related to the maintenance of the perennial life cycle; in both diploid *F. vesca* and octoploid *F*. × *ananassa*, the same environmental conditions promote both floral induction and BC development (e.g. Hytönen and Kurokura, 2020). A similar phenomenon has been recently described in the perennial *Arabis alpina*, in which floral initiation and the initiation of new AXBs take place during vernalization. In *A. alpina*, the AXBs initiated before the cold period are destined to remain dormant, while the buds initiated during and after the cold grow out as vegetative branches that are capable of flowering post-vernalization (Vayssières et al., 2020). Thus, maintaining a pool of undifferentiated dormant AXBs under floral-inducing conditions is a strategy to ensure vegetative growth the following season in herbaceous perennials including *A. alpina and F. vesca* .

## Materials and Methods

### Plant material and growing conditions

Seasonal flowering woodland strawberry (*F. vesca* L.) accession FIN56 (PI551792) and perpetual flowering *F. vesca* cultivar ‘Hawaii-4’ (PI551572) were obtained from the National Clonal Germplasm Repository, Corvallis, USA. Seeds of the perpetual flowering cultivar ‘RV’ (PI551824) were obtained from Dr Amparo Monfort (Centre for Research in Agricultural Genomics, Barcelona, Spain). Transgenic lines in FIN56 background have been previously reported (Koskela et al. 2012; Mouhu et al. 2013). FIN56 and transgenic plants in FIN56 background were clonally propagated from mother plants grown in greenhouse under LDs (18 h) at 18 ± 2°C. RV, H4, and transgenic plants in H4 background were produced from seeds that were scarified with a 1M H_2_SO_4_ solution for 5 min and soaked in water at 28°C overnight before germination. Seeds were germinated under LDs at 22°C until cotyledons were visible. Transgenic seedlings were selected based on GFP fluorescence.

Growth chambers were equipped with LED lamps (AP67, Valoya, Finland) or LED tubes, supplying 200 µmol m^–2^ s^–1^ of photosynthetic photon flux density (PPFD). In the greenhouse, natural lighting was complemented with 120 µmol m^–2^ s^–1^ PPFD from high-pressure sodium lamps (Airam 400W, Kerava, Finland) and 8 μmol m^−^^2^ s^− 1^ PPFD from incandescent lights were used to extend day length in some experiments. Experiment-specific light and temperature conditions are described in the figure legends. Plants were propagated and grown on Jiffy peat disks (Jiffy Products International BV) or in pots filled with peat (Kekkilä, Finland) and fertilized when needed. Seedlings had developed at least 2–3 leaves before environmental treatments. For the growth regulator treatments, GA3 (Duchefa) and Pro-Ca (BAS125; BASF) solutions were prepared as described in Mouhu et al. (2013) and seedlings were sprayed at 2–3 leaves stage and 2 weeks later. Phenotypic observations were performed as described in the figure legends.

### Generation of transgenic constructs and plant transformation

To produce the *FvGA20ox4*–RNAi construct, a 479-bp sequence within the first exon of gene09034 (*F. vesca* v2.0.a2; Li et al. 2018) was amplified from H4 genomic DNA using Phusion DNA polymerase (NEB, catalog #M0530S) using the primers described in Supplemental Table S3. The forward primer included the “cacc” sequence required for cloning into the pENTR™/D-TOPO Cloning Kit (Invitrogen, catalog #K240020). After cloning into pENTR and confirmation by sequencing, the fragment was cloned into the binary vector pH7GWIWG2-7F2.1 by LR Clonase (Invitrogen, catalog #11791-100). The plasmid was transformed into *Agrobacterium tumefaciens* GV3101 by electroporation.

Transformation of the RNAi construct into *F. vesca* H4 WT plants was carried out as previously described (Kang et al., 2013; Caruana et al., 2018) using cotyledons as starting material. Individual transgenic lines were identified and confirmed by GFP fluorescence and by PCR of genomic DNA using primers targeting the hygromycin gene (Supplemental Table S3).

### Expression analysis

Total RNA extraction, cDNA synthesis and real-time PCR were performed as described by Mouhu et al. (2009). The number of technical replicates per sample was three and *FvMSI1* was used as a stable reference gene for standardization. Relative expression was calculated using the ΔΔCt method (Pfaffl, 2001) unless stated otherwise in the figure legend. RT-qPCR primer sequences used were the same as in Mouhu et al. (2009) and Koskela et al. (2012), except for the *FvGA20ox4* expression analysis shown in Figure 2A, where the primers specified in Supplemental Table S3 were used. Sampling details including time points and the number of biological replicates are specified in the figure legends.

### Statistical methods

Logistic regression was used to test the main factors and either Tukey HSD or Dunnets was used for subgroup analysis. Statistics were done using R 4.0.3 (R Core Team, 2020), the stats (v3.6.2; R Core Team, 2020) and the DescTools (v0.99.40; Andri et mult. al. S, 2021) packages.

### Accession numbers

Sequence data from this article can be found in the GenBank/EMBL data libraries under accession numbers available in Supplemental Table S4.

## Supplemental data

The following materials are available in the online version of this article. 

**Supplemental Figure S1**. *FvGA20ox4* is the main *FvGA20-oxidase* controlling axillary bud fate in the perpetual flowering *F. vesca*.

**Supplemental Figure S2**. ’RV’ flowers exclusively under long days.

**Supplemental Figure S3**. ‘RV’ starts forming BCs rapidly upon exposure to long days.

**Supplemental Figure S4**. *FvGA20ox4* is the main *FvGA20-oxidase* controlling axillary bud fate in the seasonal flowering *F. vesca*.

**Supplemental Figure S5**. Photoperiod controls axillary meristem fate independently of flowering in seasonal flowering FIN56 and NOR1.

**Supplemental Figure S6**. Cool temperatures promote BC formation in seasonal flowering FIN56 and NOR1.

**Supplemental Figure S7**. *FvSOC1* is not required for stolon development at warm temperature in the seasonal flowering FIN56.

**Supplemental Table S1**. Characteristics of *F. vesca* genotypes used in this study.

**Supplemental Table S2**. Statistical analysis of axillary bud fate in H4 and RV.

**Supplemental Table S3**. Primers used in this study.

**Supplemental Table S4**. Gene IDs in *F. vesca* genome v4a2.

## Supplementary Material

kiab421_Supplementary_Data

## Notes

Senior author.

J.A., S.S., J.L., and E.A.K.: carried out the experimental work; J.C.: produced the FvGA20ox4-RNAi plant materials under the supervision of Z.L.; A.M.: supervised the experiments carried out at CRAG; T.H. and E.A.K.: supervised the experiments carried out at UH; E.A.K., J.A., and T.H.: drafted the manuscript with input from all the co-authors.

The author responsible for distribution of materials integral to the findings presented in this article in accordance with the policy described in the Instructions for Authors (https://academic.oup.com/plphys/pages/General-Instructions) is: Timo Hytönen (timo.hytonen@helsinki.fi).
