# Erratum to: Woodland strawberry axillary bud fate is dictated by a crosstalk of environmental and endogenous factors

**DOI:** 10.1093/plphys/kiab491

**Published:** 2021-11-11

**Authors:** Javier Andrés, Julie Caruana, Jiahui Liang, Samia Samad, Amparo Monfort, Zhongchi Liu, Timo Hytönen, Elli A Koskela

*Plant Physiology*, https://doi.org/10.1093/plphys/kiab421

When this paper first published, an author name was given as “Javier Andrés Jimenez”. This should have been “Javier Andrés”. This has now been corrected online.

