# Supplementary material for: Woodland strawberry axillary bud fate is dictated by a crosstalk of environmental and endogenous factors
Source: Plant Physiol. 2021 Sep 1;187(3):1221–34. doi: 10.1093/plphys/kiab421 (PMC8567079; doi:10.1093/plphys/kiab421)
Supplement: kiab421_Supplementary_Data [file kiab421_supplementary_data.pdf]

Supplemental Figures

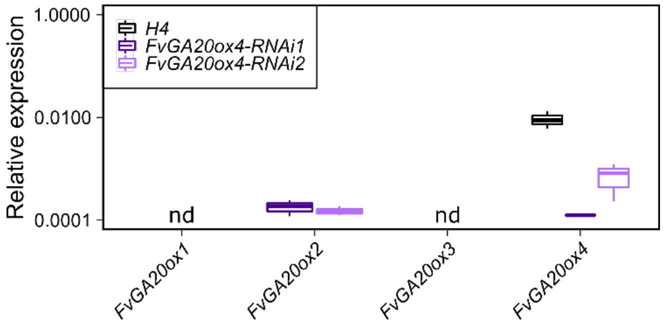

Supplemental Figure S1. *FvGA20ox4* is the main *FvGA20-oxidase* controlling axillary bud fate in the perpetual flowering *F. vesca*. Expression of all *FvGA20-oxidases* in SAM samples from *FvGA20ox4*-RNAi lines #1 and #2, and H4 wild type seedlings grown in a growth chamber with led tubes, under 12h photoperiod at 22°C. SAM samples including 6-8 apexes per biological replicate were collected when five leaves had developed. Relative expression was calculated using the ΔCt method with *FvMSI1* as reference gene (n=4). The center lines represent the median and upper and lower hinges represent the first and third quartiles. Upper and lower whiskers extend from the upper hinge to 150% of the interquartile range and from the lower hinge to -150% of the interquartile distance respectively. nd = not detected.

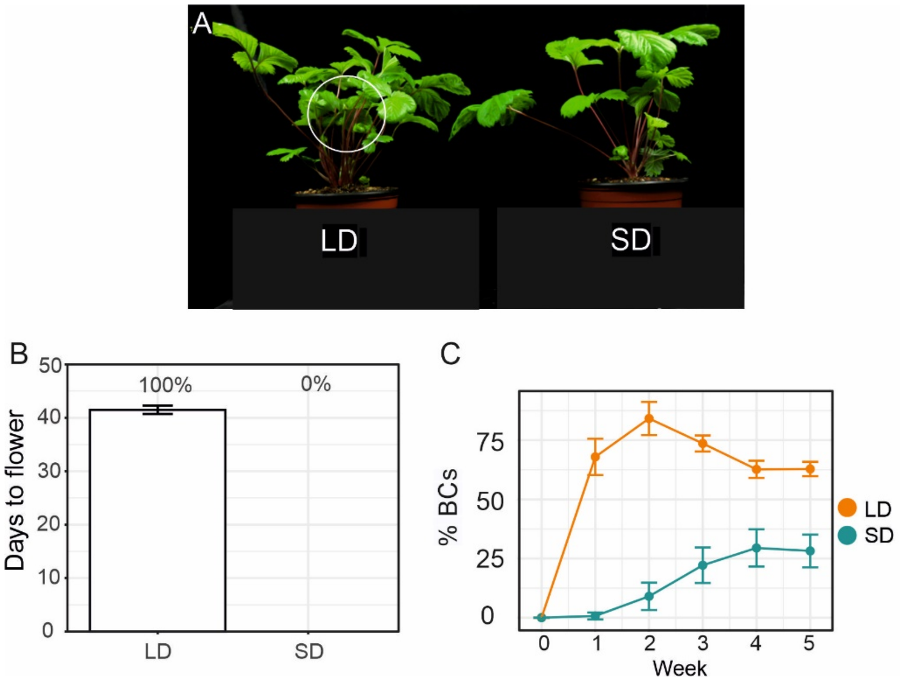

Supplemental Figure S2. ’Reine des Vallées’ flowers exclusively under long days. (A) Photograph of LD and SD grown RV seedlings on week five; (B) Flowering time and percentage of flowering plans on week seven; (C) Weekly recorded percentage of axillary buds developing branch crowns. Seedlings were germinated and grown under 8h SDs at 22°C until the beginning of the experiment. Seedlings were subjected to either 16h LDs (n = 18) or 8h SDs (n = 28) at 22°C for seven weeks. Artificial light was provided by LED tubes (Valoya, Finland; model L28, spectrum NS12). Flowering (B) was recorded weekly until the end of the experiment, and the number of BCs was recorded weekly until week five. In (C), error bars present confidence intervals. LD = long day; SD = short day; BC = branch crown.

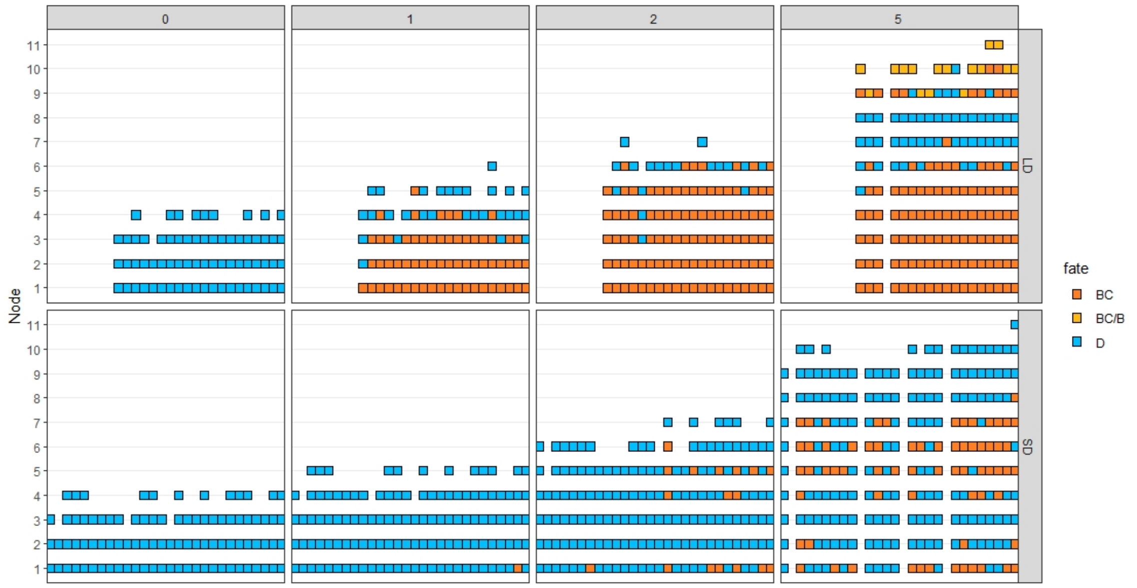

Supplemental Figure S3. ‘Reine del Vallées’ starts forming branch crowns rapidly upon exposure to long days. Plants were moved to LDs (n = 18-20) on week 0, while the SD plants (n = 24-28) remained in SD conditions for a total of five weeks at 22°C. Artificial light was provided by LED tubes (Valoya, Finland; model L28, spectrum NS12). Individual plants are plotted on the x-axis and each square represents the fate of an individual axillary bud. No flowering was observed in SD-grown plants, whereas all the LD-grown plants had visible flower buds by week five. LD = long day; SD = short day; D = dormant; BC = branch crown; BC/B = the SAM has terminated in an inflorescence and the youngest axillary bud has formed a branch crown.

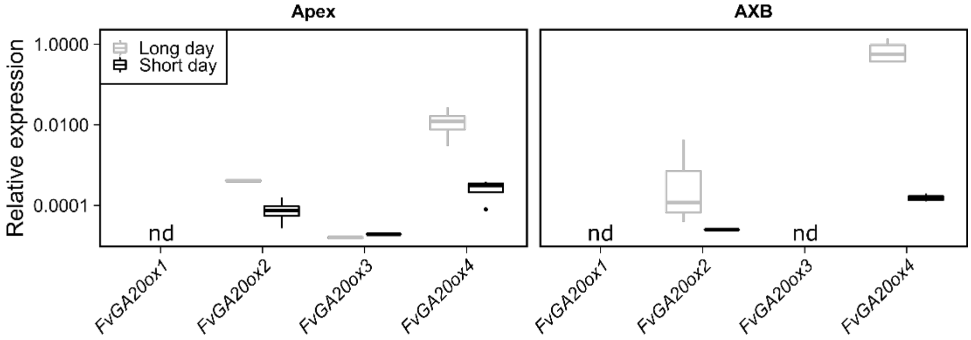

Supplemental Figure S4. *FvGA20ox4* is the main *FvGA20-oxidase* controlling axillary bud fate in the seasonal flowering *F. vesca*. Expression of all *FvGA20-oxidases* in SAM and AXB samples from stolon-propagated seasonal floweringFIN56 plants grown in greenhouse under 18 or 12h photoperiod at 18ºC. SAM and AXB samples including five apexes or AXBs per biological replicate were collected on week four. Relative expression was calculated using the ΔCt method with *FvMSI1* as reference gene (n=4). The center lines represent the median and upper and lower hinges represent the first and third quartiles. Upper and lower whiskers extend from the upper hinge to 150% of the interquartile range and from the lower hinge to -150% of the interquartile distance respectively. nd = not detected.

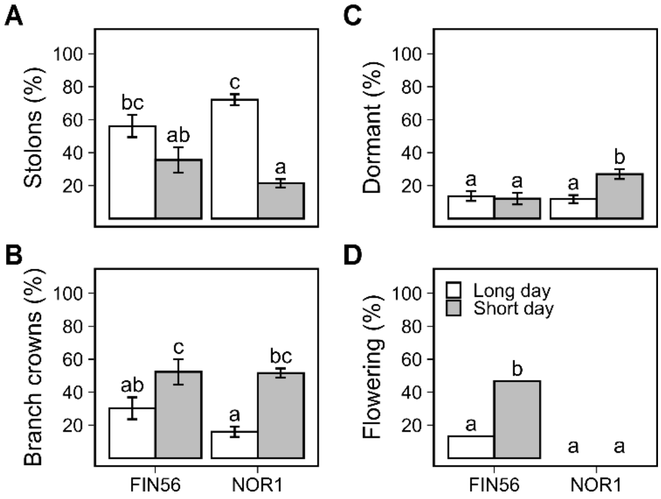

Figure S5. Photoperiod controls axillary meristem fate independently of flowering in seasonal flowering FIN56 and NOR1. Percentage of axillary buds developing stolons (A), or branch crowns (B), or remaining dormant (C). Percentage of flowering plants (D). Stolon-propagated plants were grown in chambers with led lamps under 16 and 12h photoperiod at 17ºC for five weeks; then transferred to greenhouse under 16h photoperiod at 17ºC. AXB fates were recorded up to that point, none of the plants flowered by then. Error bars represent the standard error of the mean (n=13-15) and different letters indicate significant differences calculated by logistic regression and Tukey's test ($P < 0.05$).

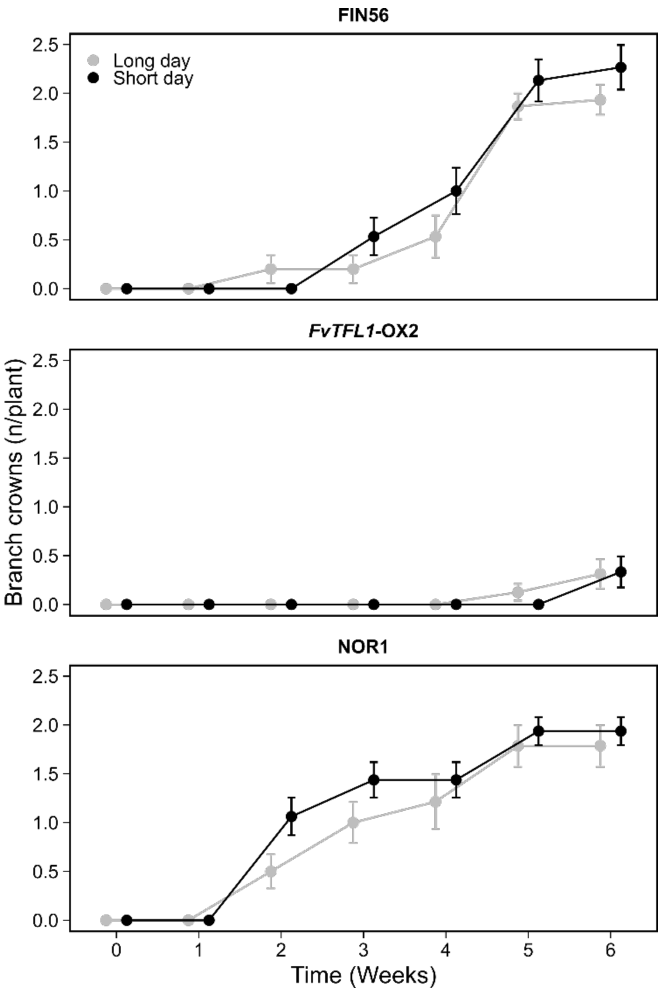

Supplemental Figure S6. Cool temperatures promote branch crown formation in seasonal flowering FIN56 and NOR1. Stolon-propagated plants of FIN56, *FvTFL1*-OX line #2 in FIN56 background, and NOR1 were subjected to photoperiod treatments (18 and 12h daylight) at 10°C for 5 weeks. Plants were grown in growth chambers equipped with LED lamps during the treatments and then transferred to a greenhouse under LDs at 18ºC. AXB observations were recorded up to week 6. Error bars represent standard error (n = 14-16).

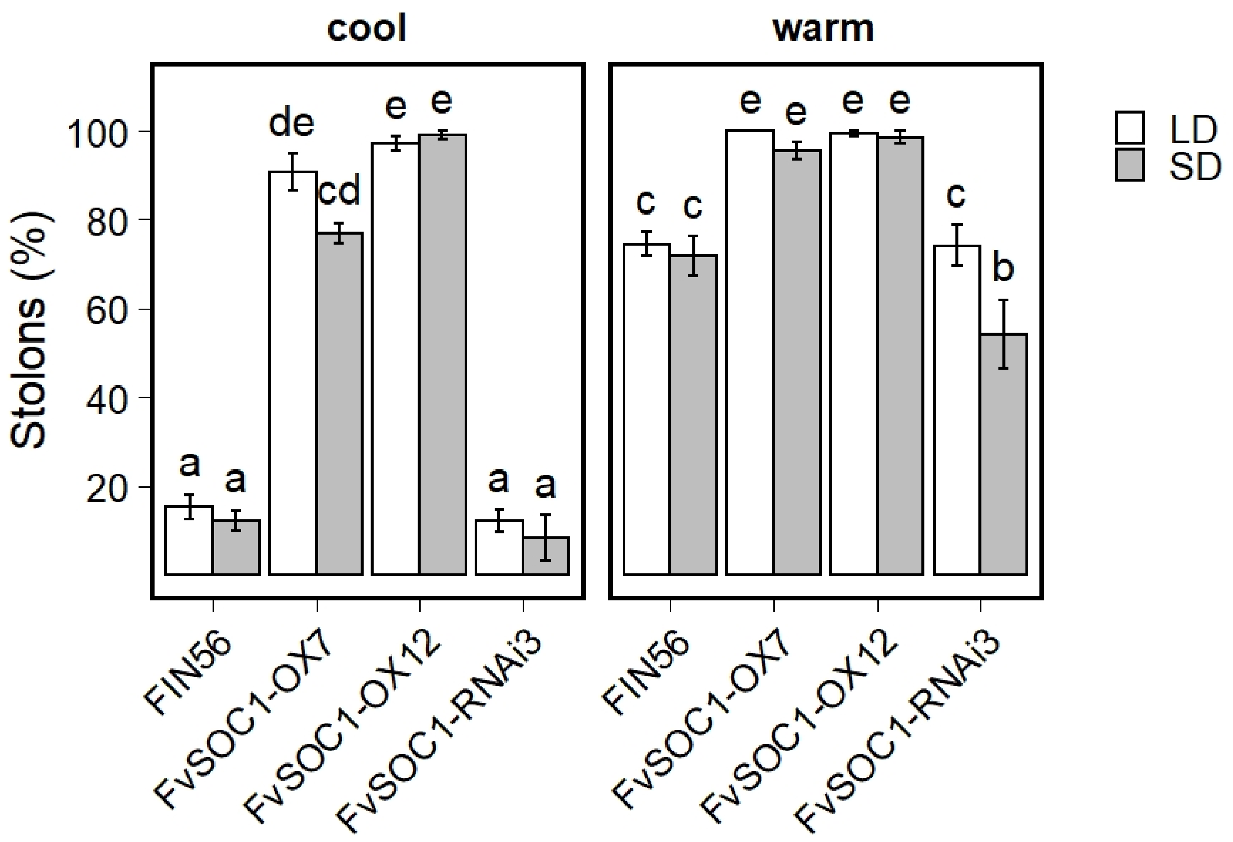

Supplemental Figure S7. *FvSOC1* is not required for stolon development at warm temperature in the seasonal flowering FIN56. Plants were grown in growth chambers equipped with LED lamps at cool (10°C) or warm (22°C) temperature under SDs (12 hour light) or LDs (18 hours of light) for five weeks, after which all the plants were moved to greenhouse (LDs, 18°C) for observations. Axillary bud fates were scored after four weeks in the greenhouse. Error bars represent the standard error of the mean (n = 9–16). Values marked by the same letter are not significantly different at $\alpha = 0.05$, calculated by logistic regression and Tukey's HSD test. LD = long day; SD = short day.

Supplemental Tables

Supplemental Table S1. Characteristics of *F. vesca* genotypes used in this study.

Genotype	Phenotype	Known mutations	Transgene	Reference
H4, Hawaii-4	Perpetual flowering (LD-induced)<br>Develops stolons	*FvTFL1* (non-functional)	na	Koskela et al. (2012)
RV, Reine des Vallées	Perpetual flowering (LD-induced)<br>Lacks stolons	*FvTFL1* (non-functional)<br>*FvGA20ox4* (non-functional)	na	This work;<br>Tenreira et al. (2017)
H4-*FvGA20ox4*-RNAi	Perpetual flowering (LD-induced)<br>Lacks stolons	*FvTFL1* (non-functional)		This work
FIN56	Seasonal flowering (SD or cool temperature-induced)<br>Develops stolons	na	na	
NOR1	Vernalization-requiring<br>Develops stolons	*FvTFL1* (mutation in the 5'-promoter)	na	Koskela et al. (2017)
FIN56-*FvSOC1*-OX	Mostly non-flowering<br>Develops stolons profusely	na	*FvSOC1* overexpression	Mouhu et al. (2013)
FIN56-*FvSOC1*-RNAi	Day-neutral flowering at 18°C<br>Reduced stolon development at 18°C	na		Mouhu et al. (2013)
FIN56-*FvTFL1*-OX	Non-flowering<br>Develops stolons	na	*FvTFL1* overexpression	Koskela et al. (2012)

na = not applicable

Supplemental Table S2. Statistical analysis of axillary bud fate in H4 and RV.

Genotype	Nodes	Fate	Treatment	Percentage	p value
RV	1 to 3	BC	Short day	73.3	
RV	1 to 3	BC	Long day	63.3	0.68
RV	1 to 3	BC	Decapitated	75.8	0.98
RV	1 to 3	D	Short day	26. 7	
RV	1 to 3	D	Long day	36. 7	0.68
RV	1 to 3	D	Decapitated	24.2	0.98
RV	4 to 5	BC	Short day	15.0	
RV	4 to 5	BC	Long day	100	8.60E-10 ***
RV	4 to 5	BC	Decapitated	81.8	7.00E-08 ***
RV	4 to 5	D	Short day	85	
RV	4 to 5	D	Long day	0	8.60E-10 ***
RV	4 to 5	D	Decapitated	18.2	7.00E-08 ***
Genotype	Nodes	Fate*	Treatment	Percentage	p value
H4	1 to 3	BC	Short day	63.3	
H4	1 to 3	BC	Long day	63.3	1
H4	1 to 3	BC	Decapitated	74.1	0.39
H4	1 to 3	S	Short day	3.3	
H4	1 to 3	S	Long day	0.0	0.28
H4	1 to 3	S	Decapitated	0.0	0.21
H4	1 to 3	D	Short day	33.3	
H4	1 to 3	D	Long day	36. 7	0.93
H4	1 to 3	D	Decapitated	25.9	0.64
H4	4 to 5	BC	Short day	5.0	
H4	4 to 5	BC	Long day	35.0	0.09
H4	4 to 5	BC	Decapitated	30. 6	0.10
H4	4 to 5	S	Short day	90.0	
H4	4 to 5	S	Long day	55.0	0.05
H4	4 to 5	S	Decapitated	66. 7	0.16
H4	4 to 5	D	Short day	5.0	
H4	4 to 5	D	Long day	10.0	0.69
H4	4 to 5	D	Decapitated	2. 8	0.91
H4	4 to 5	BC	Short day	5.0	

* BC = branch crown; S = stolon; D = dormant.

Supplemental Table S3. Primers used in this study.

Usage	5'-Forward primer	5'-Reverse primer
Amplification of the first exon of *FvGA20ox4* for generating RNAi construct	caccCATCAAAGCTTCATGCTCGACAAACC	GGATTTAAAGAAATCCACCACGACTTTCTC
Hyg-specific primers for confirming transgenesis	CAGCTTCGATGTAGGAGGGCGTG	CTATTTCTTTGCCCTCGGACGAGTG
RT-qPCR primers for *FvGA20ox4*	CCAGAGGAACTTGTTACTGAAGTAGG	TCAATTGACTGATTTGGATTCAGACTTG

Supplemental Table S4. Gene IDs in *F. vesca* genome v4a2. Gene names and accession numbers according to the last version of the *F. vesca* genome (Fragaria_vesca_v4.0.a2), available in the Genome Database for Rosaceae (http://www.rosaceae.org).

Gene name	Accession number
FvAP1	FvH4_4g29600.t1
FvGA20ox1	FvH4_7g28670.t1
FvGA20ox2	FvH4_7g12600.t1
FvGA20ox3	FvH4_7g12610.t2
FvGA20ox4	FvH4_2g35050.t1
FvGA2ox	FvH4_3g16760.t2
FvGA3ox1	FvH4_6g30780.t1
**FvGAI*	FvH4_4g34110.t1
FvMSI1	FvH4_7g08380.t2
FvSOC1	FvH4_7g12700.t5
FvTFL1	FvH4_6g18480.t1

*This gene was named *FvGAI* in Mouhu et al. (2013) and *FvRGA* in Caruana et al. (2018) and Li et al. (2018).

Supplemental Table S5. Percentage of flowering plants subjected to 10°C. Flowering in FIN56, *FvTFL1*-OX line #2 in FIN56 background, and NOR1. Plants were subjected to photoperiod treatments (18 and 12h daylight) at 10°C for 5 weeks. Flowering observations were recorded up to week 6.

Genotype	Photoperiod	Flowering
FIN56	Short days	100.00%
FIN56	Long days	86.67%
NOR1	Short days	0.00%
NOR1	Long days	14.29%
FvTFL1-OX#2	Short days	0.00%
FvTFL1-OX#2	Long days	0.00%
